# Feasibility of a Web-Based Intervention to Prevent Perinatal Depression and Promote Human Milk Feeding: Randomized Pilot Trial

**DOI:** 10.2196/32226

**Published:** 2022-05-03

**Authors:** Lacey Pezley, Lisa Tussing-Humphreys, Mary Dawn Koenig, Pauline Maki, Angela Odoms-Young, Sally Freels, Brittany DiPiazza, Felicity Cann, Kate Cares, Courtney Depa, Gintare Klejka, Manoela Lima Oliveira, Jilian Prough, Taylor Roe, Joanna Buscemi, Jennifer Duffecy

**Affiliations:** 1 Department of Kinesiology and Nutrition College of Applied Health Sciences University of Illinois at Chicago Chicago, IL United States; 2 Department of Human Development Nursing Science College of Nursing University of Illinois at Chicago Chicago, IL United States; 3 Department of Psychiatry College of Medicine University of Illinois at Chicago Chicago, IL United States; 4 Department of Epidemiology and Biostatistics School of Public Health University of Illinois at Chicago Chicago, IL United States; 5 Department of Psychology College of Science and Health DePaul University Chicago, IL United States

**Keywords:** breastfeeding, chestfeeding, perinatal, depression, anxiety

## Abstract

**Background:**

Mothers who identify as Black or African American are more likely to report depressed moods in late pregnancy and early postpartum and have the lowest rates of human milk feeding compared with all other racial groups in the United States. Internet interventions offer the potential to extend preventative and supportive services as they address key barriers, particularly for those navigating the complex and vulnerable early postpartum period. However, there is limited evidence on the feasibility of such interventions for preventing perinatal mental health disorders and improving human milk feeding outcomes in Black mothers.

**Objective:**

This pilot study aimed to assess the feasibility and preliminary findings of a web-based cognitive behavioral therapy–based internet intervention, with and without human milk feeding education and support, to prevent perinatal depression and promote human milk feeding in Black mothers.

**Methods:**

Participants were Black-identifying individuals between 20 and 28 weeks of pregnancy with human milk feeding intention and mild to moderate depressive symptoms (Patient Health Questionnaire scores 5-14). Participants were randomized to either *Sunnyside*, a 6-week cognitive behavioral therapy–based web-based intervention, or *Sunnyside Plus*, which included additional education and support to promote human milk feeding. Assessments occurred at baseline, third trimester (end of antenatal treatment), 6 weeks postpartum (end of postpartum treatment), and 12 weeks postpartum. The primary focus of this randomized pilot trial was the feasibility and preliminary outcomes of mental health and human milk feeding.

**Results:**

A total of 22 tertiary-educated participants were randomized. The mean number of log-ins was 7.3 (SD 5.3) for *Sunnyside* and 13.8 (SD 10.5) for *Sunnyside Plus*. Scores of depression and anxiety measures remained below the clinical threshold for referral to treatment in both groups. All the participants initiated human milk feeding (18/18, 100%). Most participants reported at least some human milk feeding at both 6 and 12 weeks postpartum (6/7, 86%; 11/11, 100%, or 10/10, 100%, for *Sunnyside* and *Sunnyside Plus*, respectively).

**Conclusions:**

The results suggest that tertiary-educated Black mothers at risk for perinatal depression and who intended to human milk feed were receptive to and satisfied with a web-based cognitive behavioral therapy–based internet intervention, with and without human milk feeding education and support. Preliminary findings indicate that both *Sunnyside* and *Sunnyside Plus* interventions have the potential to affect symptoms of depression, anxiety, and human milk feeding outcomes.

**Trial Registration:**

ClinicalTrials.gov NCT04128202; https://www.clinicaltrials.gov/ct2/show/NCT04128202

## Introduction

### Background

Mental health disorders are among the most common complications during pregnancy and in the first 12 months after childbirth [1-3]. Research suggests that the prevalence of perinatal anxiety disorders is at least 17% and that approximately 7% to 20% of individuals experience clinical depression at some time during the perinatal period [4-6]. Perinatal mental health disorders make it difficult to function and care for oneself and for an infant. In fact, maternal mental health is considered an important underlying factor associated with barriers and reduced rates of human milk (HM) feeding intent, initiation, exclusivity, and continuation [1-3,7].

HM feeding is considered the ideal form of infant feeding because of its extensive benefits for the lactating person and infant. All major health and professional organizations recommend exclusive HM feeding for the first 6 months of a child’s life, with continued HM feeding in combination with appropriate complementary foods for at least 1 to 2 years [8-10]. However, despite the benefits, recommendations, and high rates of intention to HM-feed, overall rates within the United States continue to be low [11]. There are many known barriers to reaching HM feeding goals, including painful or difficult latch, concerns about milk supply, lack of professional lactation support, unsupportive social and cultural norms, inadequate parental leave policies, and maternal mental health difficulties [12-16].

Certain barriers are disproportionately experienced by Black individuals, many of which stem from historical and continued oppression, systematic racism, social injustices, and structural violence. For example, Black mothers often receive limited education and differential treatment from providers regarding HM feeding information and encouragement [12]. National data show that non-Hispanic Black mothers have the lowest rates of HM feeding initiation and continuation at 6 and 12 months postpartum compared with all other racial groups in the United States. [11] In addition, Black mothers are more likely to report depressed mood in late pregnancy and early postpartum than White mothers, even after adjusting for income and education, distinguishing between the effects of race and socioeconomic status [17].

The relationship between maternal mental health and HM feeding outcomes is bidirectional; mental health disorders can make HM feeding more challenging, and difficulty with HM feeding may predict depression and anxiety [18-22]. Therefore, it is important to consider both when designing interventions to improve these outcomes. Cognitive behavioral therapy (CBT), which focuses on identifying and changing unhelpful thoughts and behaviors, has been shown to be effective in preventing perinatal depression [23]. Interventions that extend across pregnancy and postpartum and offer individualized support from professionals and peers have been shown to be successful in improving both mental health and HM feeding outcomes [24,25]. In addition, intervention components shown to improve these outcomes among Black mothers include a positive representation that enhances and normalizes HM feeding in an encouraging way, content that addresses gaps in support (eg, building a support network, advocating for oneself in the hospital, preparing for a successful return to school or work, and enhancing HM feeding self-efficacy), and professional and timely HM feeding support that continues into the postpartum period [12,26-28]. Although many effective intervention strategies exist, access to these programs can be a barrier, especially for those navigating the complex and vulnerable early postpartum period.

The internet offers great potential in extending preventative and supportive services to individuals in the perinatal period as it addresses several key barriers to success. Digital technology interventions, which include the use of web-based content and interactions, SMS text messaging, and social media, have been effective in reducing depressive symptoms and improving HM feeding outcomes [29,30]. Black mothers report that social media, for example, is a practical, convenient, and valuable way of obtaining HM feeding information and support, feeling connected with people who have overlapping lived experiences, and improving self-efficacy [31,32]. However, there is limited evidence on the feasibility of such interventions for preventing perinatal mental health disorders and improving HM feeding outcomes in Black mothers.

### Objective

The previously studied *Sunnyside* intervention is a web-based CBT-based internet program used to manage mood during the perinatal period. Findings from the pilot study showed that intervention use and satisfaction were high among participants, and symptoms of depression decreased from midpregnancy to 6 weeks postpartum [33]. To further study the relationship between mental health and HM feeding, we developed *Sunnyside Plus*, which is built upon *Sunnyside* and also includes HM feeding education and support. Therefore, the objectives of this study were 2-fold. First, we examined the feasibility of *the Sunnyside Plus* by measuring adherence to the intervention, usability, and acceptability. Second, we tested the preliminary mental health and HM feeding outcomes of *Sunnyside Plus* compared with those of *Sunnyside*.

## Methods

### Study Design and Participants

This randomized pilot trial used a comparative effectiveness research approach to compare 2 active treatments, *Sunnyside* [33] and the newly developed *Sunnyside Plus*, on maternal mental health and HM feeding outcomes among Black individuals with mild to moderate depressive symptoms upon study enrollment who intended to HM-feed their child. Although HM feeding can include the use of donor HM, in this study, HM was provided directly from the lactating parent at their breast or chest or via their expressed milk.

Participants were recruited through advertisements placed on Ovia Health [34], a nationwide web-based pregnancy forum and internet-based application, between June 12, 2020, and September 15, 2020. Inclusion criteria were as follows: (1) pregnant and between 20 and 28 weeks of gestation, (2) aged ≥18 years, (3) Black or African American, (4) intending to HM-feed their child, (5) self-reporting mild to moderate depressive symptoms (Patient Health Questionnaire [PHQ]–8 score of 5-14), (6) access to a broadband internet connection, and (7) proficiency in the English language. Exclusion criteria were as follows: (1) pregnant with multiples; (2) visual, hearing, voice, or motor impairment that would prevent completion of the study procedures; (3) diagnosed with a major depressive episode, psychotic disorder, bipolar disorder, dissociative disorder, substance use disorder, or other diagnoses for which participation in this trial was either inappropriate or dangerous based on self-report; or (4) currently receiving treatment (medication or psychotherapy) and having an intention to resume antidepressant medication after birth (ie, those who discontinued their medication during pregnancy). Those interested were directed to a brief web-based screener to assess their eligibility.

### Ethics Approval

Qualifying individuals provided electronic consent to participate. All procedures were approved by the institutional review board at the University of Illinois Chicago (UIC; IRB approval number: 2019-0519).

### Study Procedures

Following consent, qualifying individuals were immediately directed to complete the baseline assessment surveys that were provided via a REDCap (Research Electronic Data Capture; Vanderbilt University) link; study data were collected and managed using REDCap electronic data capture tools hosted by the UIC [35,36]. Participants were then randomized in a 2:1 allocation ratio to either *Sunnyside Plus* or *Sunnyside* using a block randomization method with a web-based randomization service provider, Sealed Envelope [37]. A 2:1 randomization allocation was used to gain more feasibility insights and experience with the *Sunnyside Plus* intervention component, which had not been previously studied.

All participants, regardless of group allocation, completed an initial engagement session to review the components and expectations of the study and ensure access to the treatment websites. The engagement session took place through the Cisco WebEx Meeting Center, a Health Insurance Portability and Accountability Act–compliant videoconferencing web application. Once completed, the web-based intervention began. Follow-up assessments using REDCap took place following the completion of 6 weeks of web-based lessons during pregnancy (third trimester) and at 6 and 12 weeks postpartum. A brief assessment of HM feeding continuation and exclusivity (yes or no reply) was performed on a weekly basis via SMS text messaging (SimpleTexting [38]) from 1 to 6 weeks postpartum. Participants received a US $20 Amazon gift certificate after completing each assessment.

### Interventions

#### Overview

Starting between 20 and 28 weeks of gestation, the participants began the 6-week web-based intervention (*Sunnyside* or *Sunnyside Plus*). After the birth of their baby, the intervention was continued for 6 weeks postpartum. The intervention components for each group are listed in Table 1 and described in the following sections.

**Table 1:** Overview of intervention components.

Pregnancy period and week			*Sunnyside*		*Sunnyside Plus ^a^*
**Antenatal**					
	Week 1	Part 1: Your Pregnancy and Your Mood Part 2: Worries About You and Your Baby		Part 1: HMF^b^ Benefits, Recommendations, and Safety Part 2: Learning about HMF	
	Week 2	Part 1: Mood Management Part 2: Challenging Your Thinking		Part 1: HMF Basics Part 2: HMF Positions	
	Week 3	Part 1: Stress in Pregnancy Part 2: Positive Activities in Pregnancy		Part 1: Realities of HMF Part 2: Realities of HMF (continued)	
	Week 4	Part 1: Communication and Support Part 2: Changing Relationships		Part 1: Preparing to HM^c^-feed by Building Your Support Network Part 2: Building Your Support Network (continued)	
	Week 5	Part 1: Monitoring Kick Counts and Other Pregnancy Anxieties; HMF in the Time of COVID-19 Part 2: Planning for Postpartum and Employment Issues		Part 1: Feeding and Growth Patterns of a Newborn Part 2: Expressing, Storing, and Feeding Human Milk	
	Week 6	Part 1: Preparing for Birth and After Part 2: Moving Forward and Conclusions		Part 1: HMF Immediately After Birth Part 2: Advocating for Yourself in the Hospital; HMF in the Time of COVID-19	
**Postpartum**					
	Week 1	N/A^d^		Working Through Early HMF Challenges HMF text support messages (3) Lactation support calls (at least 1)	
	Week 2	Baby Blues/Relationships with Family and Friends		HMF Challenges and Solutions HMF text support messages (3) Lactation support calls (at least 1)	
	Week 3	N/A		Feeding and Growth Patterns of a Newborn (booster) HMF text support messages (2) Lactation support calls (as needed)	
	Week 4	Relationships and Unhelpful Thoughts		Expressing, Storing, and Feeding HM (booster) HMF text support messages (2) Lactation support calls (as needed)	
	Week 5	N/A		Using Your HMF Support Network HMF text support messages (1) Lactation support calls (as needed)	
	Week 6	Thoughts and Healthy Activities		Your HMF Journey Continues HMF text support messages (1) Lactation support calls (as needed)	

^a^*Sunnyside Plus* content includes all *Sunnyside* content plus the HMF-related content listed.

^b^HMF: human milk feeding.

^c^HM: human milk.

^d^N/A: not applicable.

#### Sunnyside

The *Sunnyside* intervention is a web-based intervention (a website with didactic material and tools) targeting skills to manage mood during and after pregnancy [33]. *Sunnyside* comprises 6 weeks of web-based lessons during pregnancy and web-based booster sessions at 2, 4, and 6 weeks postpartum. The intervention website was based on CBT and interpersonal therapy principles and comprised 12 learning modules covering basic skills (eg, behavioral activation and cognitive restructuring). Tools to assist in learning and implementing skills were associated with each learning module. The Feel Tool (ie, mood rating and feelings entry) encouraged participants to rate their mood each time they visited the site to obtain a better sense of their day-to-day feelings. The Think Tool (ie, thought record) was used to track one’s thoughts and discern between helpful and harmful thinking. Participants tracked their daily behaviors, identified patterns, and planned future positive activities using the Do Tool (ie, activity scheduling or monitoring and goal setting). In this study, participants were given unlimited access to the web intervention content that comprised lessons and tools and were encouraged to use the site at least twice weekly as new modules become available (every 3-4 days). The web-based lessons that were to be completed during pregnancy required approximately 40 to 60 minutes per week for 6 weeks. The web-based lessons completed during the first 6 weeks postpartum required approximately 10 to 20 minutes per week for 6 weeks. The UIC Center for Clinical and Translational Science Technology Core was responsible for hosting and maintaining the site.

#### Sunnyside Plus

*Sunnyside Plus* is built upon *Sunnyside* but also includes additional education and support to promote HM feeding. Education and skill promotion for HM feeding was provided during the 6 weeks of web-based lessons during pregnancy and then continued through 6 weeks postpartum. This postpartum support involved weekly web-based lessons, text support messages, and video support calls with a lactation specialist. The research team requested that at least 2 lactation support calls take place; however, beyond that, support was provided on an as-needed basis determined by the participant. Importantly, the participants had the option to choose who provided lactation support from a racially diverse team. Text support messages were sent using SimpleTexting [38], a user-friendly text-marketing software. Frequency of messages tapered from 3 to 1 message per week during the first 6 weeks postpartum. The SMS text message content included HM feeding encouragement and a reminder of the web-based lactation support.

Intervention components shown to improve mental health and HM feeding outcomes among Black mothers were central to the intervention design and development. These included Black feminist thought as a theoretical foundation—an acknowledgment that Black mothers experience life at the intersection of multiple oppressions, positive and nurturing representation of Black mothers’ HM feeding, and culturally relevant professional HM feeding support across pregnancy and postpartum [26,28]. For both groups, modules specific to anxiety and HM feeding during the COVID-19 pandemic were included in the intervention content.

### Measures

#### Overview

The primary focus of this randomized pilot trial was feasibility (adherence to and satisfaction with the intervention) and preliminary outcomes on depression and anxiety symptom severity and HM feeding initiation, continuation, and exclusivity. Participants’ sociodemographic data, parity, pregnancy-related variables, HM feeding history, mental health history, and birth-related variables were also measured. The outcomes were largely assessed using standardized measures or established questions from national sources (eg, the Centers for Disease Control and Prevention [CDC] National Immunization Survey and CDC Pregnancy Risk Assessment Monitoring System questionnaire).

#### Adherence

Adherence to the web-based intervention was measured by the number of log-ins to the site during the intervention period, lessons read, and tools completed. Adherence to text and video call interactions was measured by the number of weekly text question responses and the number of lactation video calls completed within the first 6 weeks postpartum.

#### Usability and Acceptability

The Usefulness, Satisfaction, and Ease of Use (USE) questionnaire [39] was designed to measure satisfaction (eg, “It is pleasant to use.”), usefulness (eg, “It makes the things I want to accomplish easier to get done.”), ease of use (eg, “I can use it successfully every time.”), and ease of learning (eg, “It is easy to learn to use it.”) on a Likert scale ranging from 1=*strongly disagree* to 7=*strongly agree*. Higher scores indicate greater usability and acceptability.

#### Depression and Anxiety Symptoms

The PHQ-9 [40] comprises 9 scored items and 1 unscored item, which reflect overall functioning and impairment because of depressive symptoms. The PHQ-9 uses a Likert scale to determine the frequency of experienced depressive symptoms over the past 2 weeks ranging from 0=*not at all*, 1=*several days*, 2=*more days than not*, and 3=*nearly every day*. Higher values correspond to greater frequency. Scoring the PHQ-9 is simple and efficient. The measure yields only one score, which is determined by summing the positively endorsed items (1-3) at the noted values. PHQ-9 scoring interpretations are as follows: 1 to 4=minimal, 5 to 9=mild, 10 to 14=moderate, 15 to 19=moderately severe, and 20 to 27=severe depressive symptoms. Owing to the large anticipated volume of respondents to our national web-based recruitment effort, the 8-item PHQ, which does not include an assessment of suicidality, was used for eligibility screening. It would have been out of our team’s ability to efficiently contact and evaluate all persons who might have expressed suicidal intent or plan and who were not enrolled in the study.

The Inventory of Depression and Anxiety Symptoms (IDAS) [41] is a 64-item measure of depression (including a 20-item General Depression Scale) and anxiety symptoms that has been validated in postpartum mothers. The IDAS was developed specifically in response to a National Institute of Mental Health initiative to provide a more sensitive measurement of depression and its symptom dimensions (eg, dysphoria, lassitude, insomnia, suicidality, and appetite loss) for use in clinical trials. The IDAS uses a Likert scale ranging from 1=*not at all* to 5=*extremely*. The 20-item General Depression Scale was used in this study, with an overall range from 20 to 100. Higher scores represent greater depressive symptoms.

The Generalized Anxiety Disorder (GAD-7) questionnaire [42] is a 7-item measure that assesses anxiety symptom severity using a frequency Likert scale. The values of the scale are 0=*not at all*, 1=*several days*, 2=*more than half the days*, and 3=*nearly every day*. Higher values correspond to greater frequencies. The measure yields only 1 score (0-21), which is determined by summing the positively endorsed items (1-3) at the noted values. The GAD-7 interpretations are as follows: 0 to 4=minimal, 5 to 9=mild, 10 to 14=moderate, and 15 to 21=severe anxiety symptoms.

#### HM Feeding Outcomes

The Infant Feeding Practices Study 2 [43] was developed by the Food and Drug Administration in collaboration with the CDC to collect data on infant feeding practices used by US mothers. For the purposes of this project, we used the Prenatal Questionnaire to assess infant feeding intent, HM feeding knowledge, and self-efficacy.

The Prenatal Breastfeeding Self-Efficacy Scale (PBSES) was developed by Wells et al [44] in 2006 to assess perceived HM feeding self-efficacy during pregnancy. The scale comprises 20 items with ranges on a 5-point Likert-type scale from 1=*not at all sure* to 5=*completely sure*, with an overall range from 20 to 100. Higher scores indicate greater levels of prenatal HM feeding self-efficacy.

The Breastfeeding Self-Efficacy Scale–Short Form was developed by Dennis and Faux [45] to measure postpartum HM feeding self-efficacy using a theoretical framework from the Social Cognitive Theory by Bandura. The instrument has 14 items and uses a 5-point Likert-type scale, with responses ranging from 1=*not at all confident* to 5=*always confident* and overall scores ranging from 14 to 70. Higher scores indicate greater levels of HM feeding self-efficacy.

Initiation, exclusivity, and duration of HM feeding were assessed during the postpartum period using questions from the CDC National Immunization Survey. Weekly assessments of duration and exclusivity were also performed via SMS text messages.

### Statistical Analyses

Statistical analyses were performed using R software [46]. This study used a repeated measures design with 2 intervention groups (*Sunnyside* and *Sunnyside Plus*). Data were examined to assess for outliers. Descriptive statistics were obtained by computing means and SDs for continuous variables and frequencies for categorical variables. Significance testing for within- and between-group differences in mental health and HM feeding outcomes were assessed; however, this feasibility study was not powered for these types of analyses; therefore, our data are largely presented descriptively. These results should be interpreted with caution because of the small sample size. Differences in the baseline characteristics between the intervention groups were assessed using an independent 2-sample *t* test (continuous variables) and Fisher exact test (categorical variables). The intervention feasibility data, including adherence to the intervention, usability, and acceptability, were assessed using descriptive statistics.

## Results

### Eligibility Screening

**Figure 1:**
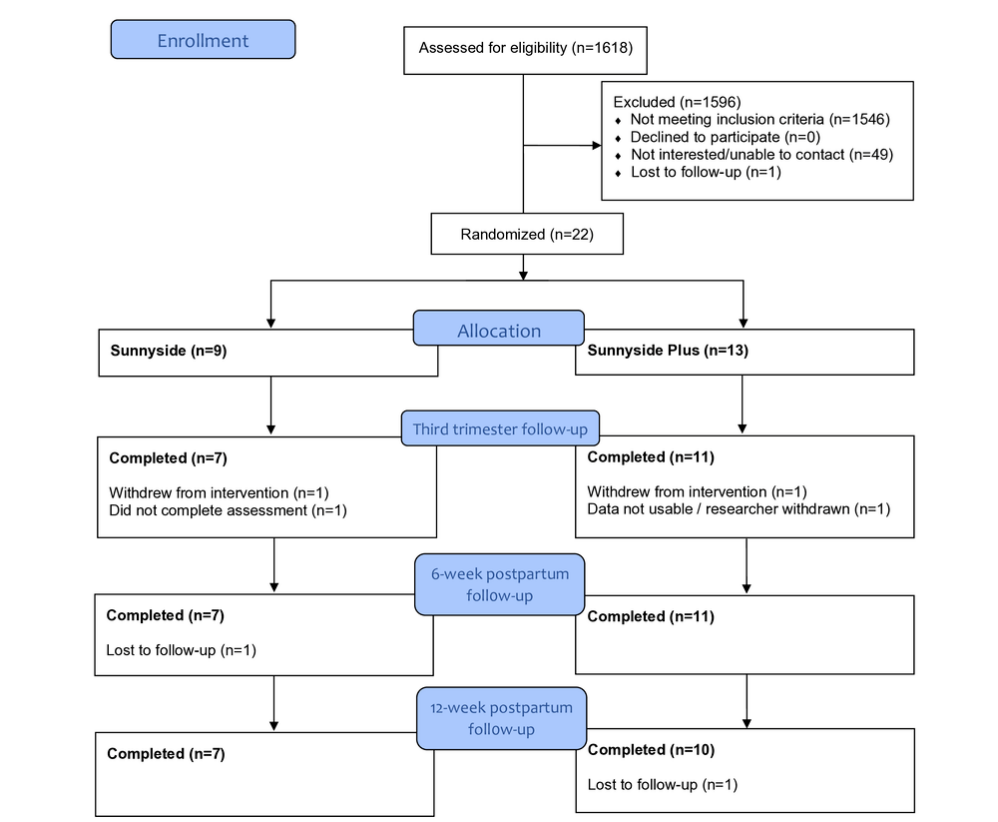
CONSORT (Consolidated Standards of Reporting Trials) flow diagram.

In total, 1618 individuals (an average of 17 per day) completed the web-based screener across a 3-month period. Of these 1618 respondents, the mean age was 30.9 (SD 3.3) years, 539 (33.3%) were identified as Black or African American, 1171 (72.3%) intended to HM-feed, and the mean PHQ-8 was 6.2 (SD 4.7). Approximately 4.45% (72/1618) of individuals met the inclusion criteria. The major factors for exclusion were race and the estimated gestational age (EGA). Of the 1618 respondents, 874 (54.01%) of respondents identified as White. Only 38.44% (622/1618) of the respondents had an EGA between 20 and 28 weeks, which was the inclusion criterion for this study. Most had an EGA of <20 weeks (971/1618, 60.01%). Of those who qualified based on the eligibility screener, 31% (22/72) individuals were randomized and received the intervention; those who were not randomized were ultimately not interested in participating, unable to contact, or lost to follow-up. Further details are provided in Figure 1.

### Participants

A total of 22 tertiary-educated Black pregnant individuals in their second trimester (mean EGA 22.6 [SD 2.5] weeks) participated in this US-based study. The mean age of the participants was 30.4 (SD 3.9) years. Most participants were married or partnered and cohabiting (17/22, 77%), employed full-time (13/22, 59%), or had private health insurance (16/22, 73%). All participants attended at least some college degree (12/22, 55%) or held a graduate or professional degree (10/22, 45%). Just over half (12/22, 55%) of the participants reported an annual household income of ≥US $51,000, with the average household size being 2.5 (SD 1). Maternal prepregnancy BMI (kg/m^2^) was calculated from self-reported height and prepregnancy body weight. When data from medical records were available, we explored the differences between self-reported and medical record data and found no differences. Over half of the participants (14/22, 64%) had obesity. The mean PHQ-9 score at baseline was 6.6 (SD 2.9), with most participants having mild depressive symptoms (15/22, 68%). The mean GAD-7 score was 6.05 (SD 4.7), with most patients having none to mild symptoms of anxiety (16/22, 73%). Most participants were nulliparous at enrollment (14/22, 64%). Regarding HM feeding–related variables, approximately one-quarter of participants reported being HM-fed themselves as an infant (6/22, 27%), most had no prior HM feeding experience (17/22, 77%), and most intended to HM-feed exclusively for at least the first few weeks postpartum (19/22, 86%). The PBSES score at baseline was 81 (SD 14.1), and the average HM feeding duration goal was 13.7 (SD 5.7) months. The participants in the intervention groups did not differ significantly in any baseline characteristics, suggesting that the random assignment–generated groups were equivalent at baseline (Table 2).

**Table 2:** Baseline sample characteristics by intervention group (N=22).

Variable		Overall	*Sunnyside* (n=9)	*Sunnyside Plus* (n=13)
Age (years), mean (SD)^a^		30.4 (3.9)	29.7 (4.7)	30.9 (3.3)
Ethnicity (non-Hispanic), n (%)		21 (96)	8 (89)	13 (100)
**Relationship status, n (%)**				
	Single	5 (23)	2 (22)	3 (23)
	Married or partnered (cohabitating)	17 (77)	7 (78)	10 (77)
Household size, mean (SD)		2.5 (1)	2.6 (1)	2.5 (1.1)
**Annual household income (US $), n (%)**				
	≤50,999	10 (45)	5 (56)	5 (38)
	≥51,000	12 (55)	4 (44)	8 (62)
**Education, n (%)**				
	Some college and 2- or 4-year college degree	12 (55)	5 (56)	7 (54)
	Graduate or professional degree	10 (46)	4 (44)	6 (46)
**Occupation, n (%)**				
	Homemaker	5 (23)	3 (33)	2 (15)
	Employed part-time	4 (18)	3 (33)	1 (8)
	Employed full-time	13 (59)	3 (33)	10 (77)
**Health insurance, n (%)**				
	Private insurance	16 (73)	7 (78)	9 (69)
	Medicaid	6 (27)	2 (22)	4 (31)
Prepregnancy BMI obese category, n (%)		14 (64)	4 (44)	10 (77)
PHQ-9^b^ moderate (10-14) category for depressive symptoms, n (%)		3 (14)	2 (22)	1 (8)
GAD-7^c^ moderate to severe (10-21) category for anxiety symptoms, n (%)		6 (27)	2 (22)	4 (31)
EGA^d^ at enrollment (weeks), mean (SD)		22.6 (2.5)	23.1 (2.9)	22.3 (2.3)
Nulliparous at enrollment, n (%)		14 (64)	4 (44)	10 (77)
Participant was HM^e^-fed as an infant, n (%)		6 (27)	2 (22)	4 (31)
No past HM feeding experience, n (%)		17 (77)	6 (67)	11 (85)
HM feeding self-efficacy (PBSES^f^), mean (SD)		81 (14.1)	77.3 (14.3)	83.5 (14)
Intend to HM-feed exclusively in the first few weeks PP^g^, n (%)		19 (86)	8 (89)	11 (85)
HM feeding goal duration (months), mean (SD)		13.7 (5.7)	12.7 (6.2)	14.3 (5.5)

^a^Participants in the intervention groups did not differ significantly on any baseline characteristics.

^b^PHQ-9: Patient Health Questionnaire-9.

^c^GAD-7: Generalized Anxiety Disorder-7.

^d^EGA: estimated gestational age.

^e^HM: human milk.

^f^PBSES: Prenatal Breastfeeding Self-Efficacy Scale.

^g^PP: postpartum.

### Attrition

Approximately 78% (7/9) of *Sunnyside* participants and 77% (10/13) of *Sunnyside Plus* participants completed the study through 12 weeks postpartum. Approximately 9% (2/22) of participants (1 from each intervention group) withdrew from the study after the baseline assessment. One of the *Sunnyside* participants did not complete the third trimester follow-up assessment but continued with the study. One of the participants in the *Sunnyside Plus* group did not engage in the web-based intervention or complete the assessments and was therefore withdrawn by the research team after the baseline assessment. Approximately 9% (2/22) of additional participants (1 from each intervention group) were considered lost to follow-up during the postpartum period; their mood scores did not differ from those who completed the study.

### Site Use

Adherence to the web-based intervention was measured by the number of log-ins to the site during the intervention period, number of lessons accessed, and number of tools completed. Table 3 shows site use data. The mean number of log-ins across the 6-week intervention plus booster sessions was 7.3 (SD 5.3) for *Sunnyside* and 13.8 (SD 10.5) for *Sunnyside Plus*. Within the *Sunnyside* group, the average number of lessons accessed during pregnancy (from a total of 13) was 10.1 (SD 3.5) and during postpartum (from a total of 3) was 1.6 (SD 1.3). Within the *Sunnyside Plus* group, the average number of lessons accessed during pregnancy (from a total of 13) was 9.7 (SD 4.1) and during postpartum (from a total of 9) was 2.6 (SD 3.3). Approximately 67% (6/9) of *Sunnyside* participants and 58% (7/12) of *Sunnyside Plus* participants completed at least 50% of the available lessons. The average number of tools used was 11 (SD 6.6) for *Sunnyside* and 25.8 (SD 27.8) for *Sunnyside Plus*. Participants in the *Sunnyside Plus* group used the activity tool more than those in the *Sunnyside* group (*P*=.03). All other site uses were similar between the groups, with no additional differences found.

**Table 3:** Adherence data (N=22).

Program activity		*Sunnyside* (n=9)	*Sunnyside Plus* (n=12)
**Total log-ins**			
	Values, mean (SD)	7.3 (5.3)	13.8 (10.5)
	Values, range	1-17	2-39
**Total days on site**			
	Values, mean (SD)	82.9 (62.4)	90.8 (53.6)
	Values, range	0-180	7-181
**Pregnancy lessons accessed^a^**			
	Values, mean (SD)	10.1 (3.5)	9.7 (4.1)
	Values, range	5-13	2-13
**Postpartum lessons accessed^a^**			
	Values, mean (SD)	1.6 (1.3)	2.6 (3.3)
	Values, range	0-3	0-9
50% completion of lessons, n (%)		6 (67)	7 (58)
**Tool: activity scheduling or monitoring^b^**			
	Values, mean (SD)	0.4 (1)	9.8 (13.1)
	Values, range	0-3	0-36
**Tool: mood rating**			
	Values, mean (SD)	3.9 (2.2)	6.3 (9.6)
	Values, range	1-8	0-35
**Tool: feelings**			
	Values, mean (SD)	2.7 (2.2)	4.9 (5.1)
	Values, range	0-5	0-17
**Tool: thought record**			
	Values, mean (SD)	3.1 (1.5)	3.4 (2.9)
	Values, range	0-6	0-10
**Tool: goal setting**			
	Values, mean (SD)	0.9 (1.2)	1.4 (3.1)
	Values, range	0-3	0-11
**Total tools used**			
	Values, mean (SD)	11 (6.6)	25.8 (27.8)
	Values, range	4-23	0-79
**Human milk feeding text question responses**			
	Values, mean (SD)	5.4 (1.1)	5.2 (1.3)
	Values, range	3-6	2-6
**Lactation support calls**			
	Values, mean (SD)	N/A^c^	2.8 (2)
	Values, range	N/A	0-7

^a^Both intervention groups were offered 13 lessons during pregnancy. *Sunnyside* intervention offered 3 lessons during the postpartum period, and *Sunnyside Plus* offered 9 lessons during the postpartum period.

^b^*P*=.03.

^c^N/A: not applicable.

### Text and Video Call Interactions

Mean number of weekly HM feeding text question responses across the first 6 weeks postpartum was 5.4 (SD 1.1) for *Sunnyside* and 5.2 (SD 1.3) for *Sunnyside Plus*. Approximately 56% (5/22) of participants in *Sunnyside* and 46% (6/13) of participants in *Sunnyside Plus* completed all 6 weekly text questions. Response rates were similar between the groups, and no differences were found. Participants in the *Sunnyside Plus* group were offered web-based lactation support. The mean number of video calls completed during the first 6 weeks postpartum was 2.8 (SD 2). The number of calls ranged from 0 to 7. One of the participants declined to receive lactation support. The results are presented in Table 3.

### Usability and Acceptability

At the third trimester follow-up (after completion of the 6-week antenatal web-based intervention), scores on USE subscales ranged from 1 (strongly disagree) to 7 (strongly agree). Mean scores for the participants in the *Sunnyside* group were 5.3 (SD 1.3) for usefulness, 5.1 (SD 2.1) for ease of use, 5.1 (SD 2.3) for ease of learning, and 4.9 (SD 1.9) for satisfaction. Mean scores for the participants in the *Sunnyside Plus* group were 4.9 (SD 1.2) for usefulness, 5.9 (SD 1.1) for ease of use, 6.2 (SD 1.1) for ease of learning, and 5.3 (SD 1.5) for satisfaction.

At 6 weeks postpartum, the scores on the USE subscales also ranged from 1 (strongly disagree) to 7 (strongly agree). Mean scores for the participants in the *Sunnyside* group were 4.9 (SD 1.4) for usefulness, 5.4 (SD 1.3) for ease of use, 5.5 (SD 1.5) for ease of learning, and 4.7 (SD 0.7) for satisfaction. For participants in the *Sunnyside Plus* group*,* mean scores were 5.1 (SD 1.4) for usefulness, 6 (SD 1.3) for ease of use, 5.8 (SD 1.3) for ease of learning, and 5.1 (SD 1.5) for satisfaction. The data are provided in Table 4. Overall, the usability and acceptability scores in this trial were slightly higher than those in previously published pilot data on *Sunnyside* [33].

**Table 4:** Usability and acceptability^a^.

Perinatal period and usability			*Sunnyside* (n=7), mean (SD)		*Sunnyside Plus* (n=11), mean (SD)
**Third trimester**					
	Usefulness	5.3 (1.3)		4.9 (1.2)	
	Ease of use	5.1 (2.1)		5.9 (1.1)	
	Ease of learning	5.1 (2.3)		6.2 (1.1)	
	Satisfaction	4.9 (1.9)		5.3 (1.5)	
**6 weeks postpartum^b^**					
	Usefulness	4.9 (1.4)		5.1 (1.4)	
	Ease of use	5.4 (1.3)		6 (1.3)	
	Ease of learning	5.5 (1.5)		5.8 (1.3)	
	Satisfaction	4.7 (0.7)		5.1 (1.5)	

^a^Usefulness, ease of use, ease of learning, and satisfaction were measured using a Likert scale ranging from 1=*strongly disagree* to 7=*strongly agree*. Higher scores indicated greater usability and acceptability.

^b^n=10 for *Sunnyside Plus.*

### Birth Outcomes

Total gestational weeks at birth, provider type, and mode of birth did not significantly differ between the groups. Mean EGA at birth was 38.7 (SD 1.3) and 38.3 (SD 1.7) for *Sunnyside* and *Sunnyside Plus* participants, respectively. A reported 71% (5/7) and 73% (8/11) of participants in *Sunnyside* and *Sunnyside Plus*, respectively, received care under an obstetrician rather than a midwife. Approximately 43% (3/7) of *Sunnyside* and 64% (7/11) of *Sunnyside Plus* participants had a cesarean birth.

### Depression and Anxiety Symptoms

Mental health outcomes are presented in Table 5. No differences between or within the groups were detected on any of the mental health outcome measures. In both intervention groups, mean PHQ-9 scores across all follow-up visits remained <10, which is the clinical threshold for referral for treatment [40]. Mean IDAS scores remained relatively consistent across all follow-up visits for *Sunnyside* and *Sunnyside Plus*. In both intervention groups, mean GAD-7 scores across all follow-up visits remained <10, which is the threshold for moderate to severe symptoms of anxiety [42].

**Table 5:** Mean mental health outcome measures at each visit and mean change from baseline to the third trimester and to 6 and 12 weeks postpartum (N=22).

Outcomes over time			*Sunnyside* (n=9)							*Sunnyside Plus* (n=13)				
			Participants, n (%)		Outcome measure, mean (SD)		Change from baseline, mean (SD)^a^		Participants, n (%)			Outcome measure, mean (SD)		Change from baseline, mean (SD)^a^
**PHQ-9^b^**														
	Baseline	9 (100)		7.3 (3.1)		N/A^c^		13 (100)			6.1 (2.7)		N/A	
	Third trimester	7 (78)		6.4 (3.9)		−0.7 (3.4)		11 (85)			7.6 (4.8)		1.4 (2.8)	
	6 weeks postpartum	7 (78)		6 (2.7)		−1.7 (4.6)		11 (85)			6.8 (3.2)		0.5 (1.6)	
	12 weeks postpartum	7 (78)		7.3 (3.9)		−0.4 (4.1)		10 (77)			6.1 (5)		−0.3 (2.8)	
**IDAS^d^**														
	Baseline	9 (100)		44.6 (8.8)		N/A		13 (100)			42.7 (11)		N/A	
	Third trimester	7 (78)		44.1 (8)		−0.3 (14.4)		11 (85)			44.5 (11.3)		0.3 (9.8)	
	6 weeks postpartum	7 (78)		44.6 (6.1)		−1.1 (7.4)		11 (85)			46.4 (11.5)		2.2 (13.6)	
	12 weeks postpartum	7 (78)		43.4 (6)		−2.3 (9.5)		10 (77)			44.9 (12.7)		−0.2 (12.4)	
**GAD-7^e^**														
	Baseline	9 (100)		5.4 (3.8)		N/A		13 (100)			6.5 (5.3)		N/A	
	Third trimester	7 (78)		5.9 (2.9)		0.9 (2.8)		11 (85)			7.5 (6)		0.2 (4.6)	
	6 weeks postpartum	7 (78)		6.7 (5.2)		0.7 (6.1)		11 (85)			6.3 (5.7)		−1.1 (4.2)	
	12 weeks postpartum	7 (78)		4.7 (3.4)		−1.3 (3.5)		10 (77)			6.4 (5.3)		−1.6 (3.4)	

^a^Estimated mean change in the difference between the baseline and follow-up means.

^b^PHQ-9: Patient Health Questionnaire-9.

^c^N/A: not applicable.

^d^IDAS: Inventory of Depression and Anxiety Symptoms.

^e^GAD-7: Generalized Anxiety Disorder questionnaire-7.

### HM Feeding

Prenatal HM feeding outcomes were examined using descriptive statistics (data not shown). The mean intended HM feeding duration at baseline (midpregnancy) was 12.7 (SD 6.2) months for *Sunnyside* participants and 14.3 (SD 5.5) months for *Sunnyside Plus* participants. Among all participants, 86% (19/22) intended to HM-feed exclusively for at least 5 to 6 months. Baseline prenatal HM feeding self-efficacy (PBSES) scores were 77.3 (SD 14.3) for *Sunnyside* participants and 83.5 (SD 14) for *Sunnyside Plus* participants. The PBSES scores increased slightly for all participants from baseline to the third trimester.

Postpartum HM feeding outcomes are shown in Table 6. All participants initiated HM feeding treatment (18/18, 100%). At 6 weeks postpartum, 57% (4/7) of *Sunnyside* and 91% (10/11) of *Sunnyside Plus* participants reported HM feeding exclusively. At 12 weeks postpartum, 57% (4/7) of *Sunnyside* and 80% (8/10) *Sunnyside Plus* participants were exclusively HM feeding. Any HM feeding at 6 weeks postpartum was reported by 86% (6/7) of *Sunnyside* and 100% (11/11) of *Sunnyside Plus* participants. Similarly, any HM feeding at 12 weeks postpartum was reported by 86% (6/7) of *Sunnyside* and 100% (10/10) of *Sunnyside Plus* participants. No differences were detected in the postpartum HM feeding outcome measures between the groups.

**Table 6:** Comparison of postpartum human milk feeding outcomes between intervention groups.

Milk feeding and pregnancy period		*Sunnyside* (n=7)	*Sunnyside Plus* (n=11)
Initiation, n (%)		7 (100)	11 (100)
**Exclusive HM^a^ feeding, n (%)**			
	6 weeks postpartum	4 (57)	10 (91)
	12 weeks postpartum^b^	4 (57)	8 (80)
**Any HM feeding, n (%)**			
	6 weeks postpartum	6 (86)	11 (100)
	12 weeks postpartum^b^	6 (86)	10 (100)
**Self-efficacy (BSES-SF^c^), mean (SD)**			
	6 weeks postpartum	45.9 (20)	48.9 (14)
	12 weeks postpartum^b^	41.3 (18.1)	53.1 (9.5)

^a^HM: human milk.

^b^n=10 for *Sunnyside Plus*.

^c^BSES-SF: Breastfeeding Self-Efficacy Scale–Short Form.

## Discussion

### Principal Findings

This study describes the feasibility and preliminary findings of a novel CBT-based internet intervention to prevent perinatal depression and promote HM feeding in Black mothers with mild to moderate depressive symptoms and intention to HM-feed. Although both active treatment groups aimed to target skills to manage mood, the newly developed *Sunnyside Plus* intervention used evidence-based practices to promote and actively support the HM feeding.

### Feasibility

Participants for this study were enrolled within a relatively short recruitment period (3 months) of indicating interest in the study intervention. Relatively low attrition rates in the *Sunnyside* group (2/9, 22%) and in the *Sunnyside Plus* group (3/13, 23%) through 12 weeks postpartum in a population of individuals within the perinatal period and who indicated mild to moderate depressive symptoms suggested adherence to the intervention. Further adherence was shown through participant interactions with the site through log-ins, lessons accessed, and tool use and with the study team through SMS text message responses and lactation support calls. Adherence to the web-based intervention indicates that Black individuals who are in the perinatal period are willing to use an individual intervention program that involves engagement with a website and interactions via SMS text messaging and video calls. To improve adherence, future research efforts using the *Sunnyside* intervention should consider adding biweekly support calls with participants to remind them of the intervention components and resolve any potential access issues. Usability scores suggested an overall positive user experience for both the pregnancy and postpartum sections of the intervention.

### Depression and Anxiety Symptoms

After completion of the intervention at both 6 and 12 weeks postpartum, no participants in this at-risk sample met the criteria for postpartum depression. This is in line with results from the previously published pilot data on *Sunnyside* [33]. Other studies have shown a 13% prevalence rate of postpartum depression among individuals in the first year postpartum [6] and a 17% prevalence rate among at-risk individuals in the absence of an intervention [47]. Overall, the levels of perinatal anxiety symptoms remained low among the participants. Given the adverse impact of perinatal mental health disorders on both the mother and infant, including reduced rates of HM feeding [3], the overall low levels of depression and anxiety symptoms among all participants were encouraging.

### HM Feeding

In the United States, 80% of mothers intend to HM-feed in some capacity, and of those, >85% intend to exclusively HM-feed for at least 3 months; however, only one-third (32%) of mothers achieve their intended HM feeding goals [48]. In this study of individuals who intended to HM-feed in at least some capacity, intended HM feeding exclusivity and duration were high among all participants at baseline. Postpartum HM feeding self-efficacy, defined as the confidence in one’s ability to effectively HM-feed, is thought to play an important role in the relationship between postpartum depression and HM feeding [19,49-52]. Not only is high self-efficacy associated with lower levels of depressive symptoms [45,50,51] but also with longer HM feeding durations [50,51]. In this study, prenatal HM feeding self-efficacy was high among all participants, and scores increased after completion of the antenatal portion of the intervention.

According to the National Vital Statistics System, the US cesarean birth rate in 2019 was 32% [53]. Overall, 56% (10/18) of the participants in this study had a cesarean birth, which is almost double the national rate. Medical interventions during birth, including cesarean birth, may make it difficult for mothers to reach their HM feeding goals [15]. Across both intervention groups, 100% (22/22) of participants initiated HM feeding. The HM feeding initiation rate among Black individuals in the United States is 60% [54]. At 6 weeks postpartum, a greater percentage of *Sunnyside Plus* participants exclusively HM-fed than those in *Sunnyside* (10/11, 91%, vs 4/7, 57%, respectively). At 12 weeks postpartum, a greater percentage of *Sunnyside Plus* participants exclusively HM-fed than those in *Sunnyside* (8/10, 80%, vs 4/7, 57%, respectively). Most participants reported at least some HM feeding at both 6 and 12 weeks postpartum (6/7, 86%; 11/11, 100%, or 10/10, 100%, for *Sunnyside* and *Sunnyside Plus*, respectively). These rates are higher than that of research showing an 81% prevalence rate of any HM feeding at 6 weeks postpartum [51]. High rates of exclusive and continued HM feeding at 6 and 12 weeks further underscore the positive clinical impact of both intervention groups.

### Strengths and Limitations

There are several strengths of this study. The design of the intervention offers a novel approach for preventative and supportive care within the perinatal period, one that extends across pregnancy and postpartum, involves various interface options (ie, website, text, and videoconferencing), and acknowledges the logistical challenges of physically seeking care as parents of a newborn. In addition, participants consistently used all aspects of the intervention in both pregnancy and the postpartum period, suggesting interest in and satisfaction with this design of care. To reduce the race-mediated power differential, the lactation specialist team included Black and White individuals. When support was provided by a White lactation specialist, we acknowledge that race-of-interviewer effects may have been present.

This study had several limitations. Although the recruitment response was high, with an average of approximately 17 respondents each day during the 3-month recruitment period, the use of a convenient internet sample may have led to a bias toward higher education levels. In addition, our recruitment method through Ovia Health might not have been the best route, given the characteristics of those using the platform, and future projects should consider other recruitment routes. The major factors for exclusion were race and EGA. Future recruitment efforts should target internet-based applications used by those who identify as Black or African American. In addition, a system that allows for future rescreening might capture those who meet all inclusion criteria, except for the current EGA. As a small pilot study, this trial was not powered to reliably detect small significant differences or associations. The results should be interpreted with caution, and a larger trial is needed to verify these outcomes. Furthermore, the primary outcome data were based on self-report assessments, which may introduce recall bias. Finally, with no true control group, we relied on outside data to compare the rates of mental health and HM feeding outcomes.

### Conclusions

The results of this study suggest that tertiary-educated Black mothers at risk for perinatal depression and who intended to HM-feed were receptive to, engaged with, and satisfied with a web-based CBT-based internet intervention, with and without HM feeding education and support, spanning from midpregnancy through 6 weeks postpartum. Preliminary findings indicate that both *Sunnyside* and *Sunnyside Plus* interventions have the potential to affect symptoms of depression, anxiety, and HM feeding outcomes. Future studies should include a larger sample size and a longer follow-up period to better understand the differences between groups and examine the continued impact across the postpartum period.

## Appendix

Multimedia Appendix 1 CONSORT-eHEALTH checklist (V 1.6.1).

## Abbreviations

CBT: cognitive behavioral therapy
CDC: Centers for Disease Control and Prevention
EGA: estimated gestational age
GAD-7: Generalized Anxiety Disorder
HM: human milk
IDAS: Inventory of Depression and Anxiety Symptoms
PBSES: Prenatal Breastfeeding Self-Efficacy Scale
PHQ: Patient Health Questionnaire
REDCap: Research Electronic Data Capture
UIC: University of Illinois Chicago
USE: Usefulness, Satisfaction, and Ease of Use
